# Determinants of Adherence to the Mediterranean Diet: Findings from a Cross-Sectional Study in Women from Southern Italy

**DOI:** 10.3390/ijerph16162963

**Published:** 2019-08-17

**Authors:** Andrea Maugeri, Martina Barchitta, Valerio Fiore, Giuliana Rosta, Giuliana Favara, Claudia La Mastra, Maria Clara La Rosa, Roberta Magnano San Lio, Antonella Agodi

**Affiliations:** Department of Medical and Surgical Sciences and Advanced Technologies “GF Ingrassia”, University of Catania, Via S. Sofia 87, 95123 Catania, Italy

**Keywords:** diet, nutrition, socio-economic status, exercise, public health

## Abstract

The Mediterranean diet (MD)—the dietary pattern usually consumed by Mediterranean populations—can help promote a favorable health status and better quality of life. Uncovering the main factors associated with the adherence to MD may be useful in understanding and counteracting the global shift toward a Western diet, which has been documented also in the Mediterranean region. Here, we evaluated the adherence to MD and its major social and behavioral determinants in women from Catania, Southern Italy. This cross-sectional study included 841 women, aged 25–64 years, with no history of severe diseases. Adherence to MD was assessed by the Food Frequency Questionnaire and Mediterranean Diet Score (MDS). Associations between variables were tested by multivariable logistic regression analysis and expressed as an odds ratio (OR) with a 95% confidence interval (CI). Among social factors, medium and high educational levels were associated with an ideal intake of alcohol (OR = 4.059; 95%CI = 1.311–12.570; *p* = 0.015; OR = 4.258 95%CI = 1.068–16.976; *p* = 0.040; respectively), living in a couple with ideal intake of cereals (OR = 2.801 95%CI = 1.188–6.602; *p* = 0.018), and having children with an ideal intake of fruits (OR = 3.149; 95%CI = 1.245–7.762; *p* = 0.015). With respect to behaviors, current smoking was negatively associated with an ideal intake of meat (OR = 0.449; 95%CI = 0.0220–0.917; *p* = 0.028), while more engagement in physical activity was associated with an ideal intake of vegetables (OR = 6.148; 95%CI = 1.506–25.104; *p* = 0.011) and legumes (OR = 5.832; 95%CI = 1.414–24.063; *p* = 0.015). In line with these findings, moderately or highly physically active women were more likely to show medium or high adherence to MD than those who performed less physical activity (OR = 6.024; 95%CI = 1.192–30.440; *p* = 0.040; OR = 9.965 95%CI = 1.683–58.993; *p* = 0.011; respectively). Our results confirm an urgent need for public health strategies, which should take into account determinants of diet quality. Particularly, our study indicates that more engagement in physical activity is a major positive determinant of the adherence to MD.

## 1. Introduction

In the last decades, several epidemiological, population-based, and randomized clinical trials have provided evidence that the adherence to a dietary pattern rich in healthy foods—such as fruit, vegetables, whole grains, and fish—can reduce the risk of many non-communicable diseases [1,2,3]. The majority of these studies followed the approach of assessing the association between single nutrients or food groups and the occurrence of disease [4,5,6,7]. However, this approach has several limitations, such as the fact that people eat a complex mixture of foods and nutrients that may interact synergistically or antagonistically [8]. Therefore, nutritional research is shifting to the analysis of dietary pattern as a whole, with an increasing number of studies being done by summing foods considered to be important for health [9]. In this scenario, the Mediterranean diet (MD)—the dietary pattern usually consumed among the populations bordering the Mediterranean Sea—has been widely reported to contribute to a favorable health status and a better quality of life [1]. The first evidence of the beneficial effects of the MD was uncovered by the comparison of disease rates between Mediterranean and other European countries [10]. Indeed, greater adherence to MD—estimated a priori using dietary scores on the basis of the characteristic components of the traditional diet of the Mediterranean area—has been found to be associated with a reduced risk of overall mortality, cardiovascular mortality, cancer incidence and mortality, and incidence of Parkinson’s disease and Alzheimer’s disease [11,12]. Despite this evidence, we are currently witnessing a shift from the MD to a Western diet, to such an extent that a nutrition transition issue has also emerged for Mediterranean populations [6]. Therefore, public health researchers and professionals are strongly motivated to identify social and behavioral factors associated with adherence to MD, which in turn might help tailor nutritional strategies and interventions in a more focused and efficient manner. In general, it has been suggested that diet quality follows a socioeconomic gradient in terms of education, employment and income: specifically, people with higher socio-economic status consume more vegetables, fruit, whole grains, fish, and low-fat products, while those with lower socio-economic status consume more refined grains and added fats [13]. Moreover, people who follow unhealthy behaviors, such as smoking and a sedentary life, tend to consume more fast-food products and fewer fruits and vegetables [14,15,16,17].

To meet the growing need to uncover the main factors associated with MD adherence, we performed a cross-sectional study on adult women recruited in Catania, Southern Italy. We focused on women since they have a crucial role in food choice by providing meals for their families and making up the majority of the workforce in food-related jobs, health care and education. Our aim was to assess major social and behavioral determinants of adherence to MD in general, and the consumption of its specific food components in particular.

## 2. Materials and Methods 

### 2.1. Study Design

The current cross-sectional study recruited women who were referred for routine physical examination to three clinical laboratories in Catania (Italy) from 2010 to 2017. In particular, we selected non-pregnant women, aged 25–64 years, with a complete assessment of social and behavioral characteristics and anthropometric measures, and no history of cancer, CVD, diabetes, neurodegenerative and autoimmune diseases. The study protocol was approved by the Ethics Committees of the involved institutions, and the study was conducted in agreement with the Helsinki declaration. All women gave their signed informed consent to participate in the study.

### 2.2. Data Collection

Data were collected by trained interviewers using structured questionnaires. Age was categorized according to tertile distribution as follows: 1st tertile (25–33 years; *n* = 276), 2nd tertile (34–46 years; *n* = 289) and 3rd tertile (47–65 years; *n* = 276). Educational level was categorized as low (primary education or apprenticeship), medium (secondary education), or high (tertiary education). Marital status was categorized into living alone (including single, divorced or widowed) or living in couple (including married and other relationships). Employment status was categorized into employed (including full-time or part-time employment) or unemployed (including retired). Smoking status was categorized as current, former or never smoking. Body weight and height were measured using standard procedures [18,19]. BMI, calculated as weight in kilograms divided by height in meters squared, was classified as underweight (BMI < 18.5 kg/m^2^), normal weight (18.5 kg/m^2^ ≤ BMI < 25 kg/m^2^), overweight (25 ≤ BMI< 30 kg/m^2^) or obese (BMI ≥ 30 kg/m^2^), according to the World Health Organization criteria [20]. Physical activity was assessed using the long form of the International Physical Activity Questionnaire (IPAQ-L) [21] and categorized as poor (no moderate or vigorous activity), intermediate (1–149 min/week moderate, 1–74 min/week vigorous or 1–149 min/week moderate + vigorous), or ideal (≥150 min/week moderate, ≥75 min/week vigorous or ≥150 min/week moderate + vigorous), according to the American Heart Association criteria [22].

### 2.3. Dietary Assessment

Dietary data were collected using a 95-item semi-quantitative Food Frequency Questionnaire (FFQ), which was referred to the previous month [18,19,23,24,25,26,27]. FFQ was adapted from a 46-item FFQ validated for the assessment of folate intake in Italian women of child-bearing age [23]. During the interview, women were asked to indicate their frequency of consumption (classified into 12 categories from “almost never” to “two or more times a day”) and serving size (low, medium or large). The medium serving size was described by standard weight or volume measures commonly consumed in the Italian population, while small and large serving sizes were half a medium serving size or 1.5 times or more larger than a medium serving size, respectively. An indicative photograph atlas was used to estimate the amount of each food item and to minimize inaccuracies. Food intakes were calculated by multiplying the frequency of consumption by the daily portion size of each food group and adjusted for total energy intake using the residual method [28].

### 2.4. Mediterranean Diet Score

The adherence to MD was evaluated using the Mediterranean Diet Score (MDS) [29,30], based on the ideal/poor consumption of nine food categories: fruits and nuts, vegetables, legumes, cereals, lipids, fish, dairy products, meat products, alcohol and the ratio of unsaturated to saturated lipids. For vegetables, legumes, fruits and nuts, cereals, fish and the ratio of unsaturated to saturated lipids, women whose consumption was below or equal to the median value of the population were assigned a value of 0, and a value of 1 was assigned otherwise. For dairy and meat products, women whose consumption was below the median were assigned a value of 1, and a value of 0 was assigned otherwise. With respect to alcohol, a value of 1 was given to women consuming 5 to 25 g per day. Thus, MDS ranged from 0 (non-adherence) to 9 (perfect adherence), and the adherence was categorized as follows: low adherence (MDS range: 0–3), medium adherence (MDS range: 4–6), or high adherence (MDS range: 7–9) [31].

### 2.5. Statistical Analysis

Statistical analyses were performed using the SPSS software (version 21.0, SPSS, Chicago, IL, USA). Continuous variables were tested for normality using the Kolmogorov–Smirnov test, expressed as median (interquartile range, IQR), and compared using the Mann–Whitney U test or Kruskal–Wallis test. Categorical variables were expressed as frequency (percentage), and compared using the Chi-square test. Logistic regression models were applied to identify independent determinants of the ideal consumption of each food category and of medium-to-high adherence to MD. The models included both social (i.e., age groups, educational level, employment status, and having children) and behavioral (i.e., smoking status, use of supplements, physical activity level, and BMI) characteristics. Results were expressed as odds ratios (ORs) and 95% confidence intervals (CIs). All statistical tests were two-sided, and *p*-values < 0.05 were considered statistically significant.

## 3. Results

### 3.1. Characteristics of Study Population

The present cross-sectional study included 841 women, aged 25–64 years, with a complete assessment of dietary information. In brief, approximately half of women lived in a couple (50.6%), and 69.0% had at least one child. Moreover, about a third reported a low educational level (35.7%), while 55.3% were unemployed. With respect to behavioral factors, 34.2% were current smokers and 17.3% were poorly physical active. According to BMI (mean = 23.87; SD = 4.69), nearly a third of women (32.5%) were overweight or obese. We also observed that 15.4% of women were in menopause, while 15.5% reported the use of folic acid supplements.

### 3.2. Determinants of Ideal Consumption of Cereals, Fruits and Vegetables, Legumes and Fish 

We first evaluated the characteristics of recruited women according to their consumption of food categories that positively characterized the MD (i.e., cereals, vegetables, fruits and nuts, legumes, fish and the ratio of unsaturated to saturated lipids) (Table 1). Specifically, women with an ideal consumption of cereals were more likely to be unemployed (*p* = 0.030), to live in a couple (*p* = 0.002), to use supplements (*p* = 0.010), to have children (*p* = 0.010) and to have a reported higher BMI (*p* = 0.028) than those with poor consumption. Logistic regression analysis, including all social and behavioral characteristics, demonstrated that living in a couple was associated with an ideal intake of cereals (OR = 2.801 95%CI= 1.188–6.602; *p* = 0.018). Instead, women with an ideal consumption of vegetables were older (*p* < 0.001), with more women between 34–46 and 47–65 years old (*p* < 0.001) in this category. They were also less likely to be unemployed (*p* = 0.029) and current smokers (*p* = 0.019) and more likely to live in a couple (*p* = 0.001) than those with poor consumption. Interestingly, they also reported a higher BMI than their counterpart (*p* = 0.001). Logistic regression analysis revealed that, although non-significant in univariate analysis, more engagement in physical activity was the only determinant of an ideal consumption of vegetables (OR = 6.148; 95%CI = 1.506–25.104; *p* = 0.011). Women with an ideal consumption of fruits and nuts were older (*p* = 0.004), with more women between 47–65 years old (*p* = 0.027). They were also more educated (*p* = 0.040), less likely to be unemployed *(p* = 0.039) and more likely to perform physical activity (*p* = 0.037) than those with poor consumption. However, having children was the only positive determinant of the ideal consumption of fruits (OR = 3.149; 95%CI = 1.245–7.762; *p* = 0.015), when all characteristics were included in the logistic regression model. Similarly, women with an ideal consumption of legumes were older (*p* < 0.001), with more women between 47–65 years old (*p* < 0.001). They were also more likely to live in a couple (*p* = 0.001) and to have children (*p* = 0.011) and less likely to smoke tobacco (*p* = 0.038). They also reported a higher BMI than those who consumed less legumes (*p* = 0.007). Logistic regression analysis revealed that, although non-significant in univariate analysis, more engagement in physical activity was the only determinant of an ideal consumption of legumes (OR = 5.832; 95%CI = 1.414–24.063; *p* = 0.015). Univariate analysis also showed that women with an ideal consumption of fish were older (*p* < 0.001), with a higher proportion of women between 47–65 years old (*p* < 0.001). They were also more educated (*p* = 0.006), less likely to be unemployed (*p* = 0.006) and current smokers (*p* = 0.040) and to have children (*p* = 0.030) than those with poor consumption of fish. However, none of these characteristics was associated with the consumption of fish in multivariable analysis.

### 3.3. Determinants of Ideal Consumption of Meat, Dairy Products, Alcohol and Lipids

With respect to foods that negatively characterized the MD (Table 2), women with an ideal consumption of meat were older (*p* < 0.001) and less likely to smoke tobacco (*p* = 0.050) than those with poor consumption. Particularly, we observed a higher proportion of women between 47–65 years old among those with an ideal consumption of meat *(p* < 0.001). By contrast, women with an ideal consumption of dairy products were younger than those with poor consumption (*p* = 0.012), with a higher proportion of women between 25–33 years old (*p* = 0.029). Logistic regression analysis revealed that smoking tobacco was negatively associated with an ideal consumption of meat (OR = 0.449; 95%CI = 0.0220–0.917; *p* = 0.028). Instead, none of the social or behavioral factors was associated with an ideal consumption of dairy products in multivariable analysis. We also found that women with an ideal consumption of alcohol were less likely to be obese (*p* = 0.006) and in menopause (*p* = 0.030) than those with poor consumption. However, logistic regression analysis demonstrated that being moderately or highly educated was a positive determinant of ideal consumption of alcohol (OR = 4.059; 95%CI = 1.311–12.570; *p* = 0.015; OR = 4.258; 95%CI = 1.068–16.976; *p* = 0.040; respectively). Finally, we observed that women with an ideal ratio of unsaturated to saturated fatty acids were less educated (*p* = 0.020), more likely to have children (*p* = 0.013) and had a higher BMI (*p* = 0.037) than those with a poor ratio. However, none of these characteristics was associated with the ratio of unsaturated to saturated fatty acids in multivariable analysis.

### 3.4. Determinants of Adherence to Mediterranean Diet

We next evaluated adherence to MD using the MDS (mean = 4.2; range = 0–8), which allowed us to identify 33.8% women with low adherence to MD (MDS ≤ 3), 56.8% with medium adherence (3 < MDS < 7), and 9.4% with high adherence (MDS ≥ 7). Table 3 displays the characteristics of the study population according to the adherence to MD. Particularly, we observed that adherence to MD increased with increasing age, and hence women with high adherence to MD were older (*p* < 0.001). Specifically, women with high adherence to MD were more likely to be 47–65 years old than those with medium or low adherence (*p* < 0.001). Moreover, women with high adherence to MD were more educated (*p* < 0.001), less likely to be unemployed (*p* = 0.017) and more likely to live in a couple (*p* = 0.017). With respect to behavioral determinants, people with high adherence to MD were less likely to smoke tobacco (*p* < 0.001). Moreover, they exhibited a lower BMI than those who adhered less to MD (*p* < 0.001), which resulted in a lower prevalence of overweight and obesity (*p* < 0.001). However, multivariable logistic regression analysis demonstrated that only the engagement in physical activity was associated with moderate to high adherence to MD (OR = 5.500; 95%CI = 1.293–18.575; *p* = 0.031).

## 4. Discussion

In this cross-sectional study, we first pointed out several social and behavioral factors associated with the ideal consumption of specific foods and/or with adherence to MD. However, when all factors were evaluated in multivariable models, more engagement in physical activity was the only positive determinant of the ideal consumption of vegetables and legumes and of high adherence to MD in general. 

There is a general consensus that people who perform more physical activity tend to be healthier than their sedentary counterparts [32], probably due to the more varied diet consumed by physically and socially active people [33]. Instead, it has been demonstrated that sedentary individuals consume more fast food products and fewer fruits and vegetables [14,15]. Although a direct relationship between physical activity and food choices has not been yet clarified, it has been well established that a lack of exercise and an unhealthy diet tend to coexist among individuals [34]. 

Similarly, we found that current smoking was a negative determinant of the ideal consumption of meat products, which means that people who smoked tobacco reported a higher intake of red and processed meats. This is in line with previous evidence that smokers had an unhealthier diet than non-smokers [16,17,35]. Specifically, a meta-analysis of 51 studies demonstrated that people who smoked tobacco had a higher intake of energy, total and saturated fat and cholesterol and a lower intake of vitamins and fiber than non-smokers [36]. Notably, the coexistence of these unhealthy behaviors may exacerbate their deleterious effects on health, increasing the risk of non-communicable diseases [36]. The positive side of the coin is that changes in one health behavior might promote changes in overall lifestyle [37]. For instance, a study of approximately 500 smokers demonstrated that reducing and quitting smoking were associated with an increased intake of fruits and vegetables and more engagement in physical activity [37]. Likewise, the participation in programs aimed at promoting physical activity has been found to influence other important healthy behaviors, including smoking cessation [38,39,40] and dietary changes [41,42].

Among social determinants, a widely used indicator of socio-economic status is education level, because such information is often available in various national and international studies. In our study, after adjusting for other factors, having a medium or high educational level was the only positive determinant of an ideal consumption of alcohol. This partially supported the current notion that healthy dietary habits are more common among high-educated individuals, since they have more knowledge about the benefits and risks of their food choices [43]. Indeed, education might affect several health outcomes through its influence on lifestyles (e.g., physical activity, smoking habits, and diet), problem-solving capacity and values (e.g., awareness of preventive behaviors) [44,45]. Other social characteristics, such as household size and composition, are rarely addressed in this field of research. In our study, living in a couple was associated with an ideal consumption of cereals, while having children was the only positive determinant of an ideal consumption of fruits. Although these findings indicated a healthier diet among those who were members of large families, an increasing household size also means more mouths to feed and increased expenditure on food, which in turn might reduce diet quantity and variety [46]. However, our research—focusing on women and mothers—confirmed their awareness about healthy food choices for themselves and their families. Thus, further research should be encouraged to understand whether the relationships between household size, composition, and diet quality might be affected by the role of each member in their family.

Our study has several strengths. To the best of our knowledge, it is the first study examining the effect of social and behavioral determinants of adherence to MD among women from Southern Italy. Moreover, data were collected using standard and validated tools, and the socioeconomic information covers different aspects of the social status contributing individually to the relationship with MD. Finally, the majority of our results are robust, as they have been obtained after adjusting for total energy intake and by using logistic regression models. However, our study also has some limitations. Its cross-sectional design does not allow us to assess the temporality and causality of observed relationships. With respect to dietary assessment, data were collected using FFQs, which did not preclude potential measurement errors and may suffer from inaccuracies. However, other widely used tools for assessing dietary data, such as weighted records and 24-hour recalls, are prone to a degree of misreporting [47]. Thus, the administration of FFQs still remains a widely used tool for dietary assessment in epidemiological studies [47]. Finally, we cannot completely exclude the effect of unmeasured residual factors, such as household income, food security and food access.

## 5. Conclusions

In conclusions, our study shows that adherence to MD is relatively low in Southern Italy, confirming that nutrition transition is also emerging for Mediterranean populations. This reflects an urgent need for public health strategies, which should take into account determinants of diet quality. Particularly, our study indicates that more engagement in physical activity is a major positive determinant of the adherence to MD. By contrast, the coexistence of sedentary behaviors with unhealthy food choices exacerbates their deleterious effects on health. The positive aspect, however, is that is the promotion of changes in one health behavior could lead to an overall improvement of lifestyle.

## Figures and Tables

**Table 1:** Characteristics of women according to consumption of cereals, fruits, vegetables, legumes, and fish ^a^.

Characteristics	Cereals			Vegetables				Fruits		Legumes			Fish		
	Poor	Ideal	*p*-Value ^b^	Poor	Ideal	*p*-Value ^b^	Poor	Ideal	*p*-Value ^b^	Poor	Ideal	*p*-Value ^b^	Poor	Ideal	*p*-Value ^b^
Age, years	43(14)	44(13)	0.876	43(14)	44(13)	**<0.001**	43(13)	44(14)	**0.004**	43(14)	44(14)	**<0.001**	43(14)	44(12)	**<0.001**
1st tertile (25–33 years)	35.1%	30.6%	0.148	39.8%	25.9%	**<0.001**	35.4%	30.3%	**0.027**	38.6%	27.3%	**<0.001**	37.8%	27.9%	**<0.001**
2nd tertile (34–46 years)	31.3%	37.4%		35.5%	33.3%		36.1%	32.6%		36.7%	32.2%		37.1%	31.7%	
3rd tertile (47–65 years)	33.7%	32.0%		24.8%	40.9%		28.5%	37.1%		24.8%	40.6%		25.1%	40.4%	
Educational level															
Low	32.9	38.4	0.110	38.6	32.8	0.210	39	32.4	**0.040**	37.6	33.8	0.518	40.9	30.5	**0.006**
Medium	45.3	44.8		42.9	47.3		44.7	45.4		43.4	46.6		40.9	49.2	
High	21.7	16.8		18.6	20		16.3	22.2		18.9	19.6		18.2	20.3	
Employment status (% unemployed)	51.6	59	**0.030**	59	51.5	**0.029**	58.9	51.8	**0.039**	53.6	56.9	0.350	60	50.6	**0.006**
Marital status (% living in couple)	42.8	56.3	**0.002**	44.6	58.9	**0.001**	49.3	51.9	0.540	44.3	59.4	**0.001**	50.3	50.9	0.359
Smoking status															
Never smokers	53.2	58.2	0.300	54.3	57.1	**0.019**	52.9	58.5	0.085	52.7	58.6	**0.038**	55.6	55.8	**0.040**
Former smokers	11.2	9		7.9	12.4		9.3	10.9		9	11.2		7.7	12.5	
Current smokers	35.6	32.8		37.9	30.5		37.8	30.6		38.3	30.1		36.7	21.7	
Use of supplements (% users)	10.9	18.8	**0.010**	15	16.1	0.730	16.5	14.2	0.460	15.6	15.2	0.890	15	16.1	0.737
Having children (% yes)	65.9	71.2	0.190	66.9	71.9	0.220	66.9	71.2	0.290	64.6	75	**0.011**	72.6	63.8	**0.030**
Number of children	2(1)	2(1)	0.052	2(1)	2(1)	0.550	2(1)	2(1)	0.420	2(1)	2(1)	0.133	2(2)	2(1)	0.836
Body Mass Index. kg/m^2^	23.4(4.5)	23.1(5.5)	**0.028**	22.9(4.5)	23.7(5.1)	**0.001**	23.4(5.6)	23.05(4.6)	0.820	23.1(4.7)	23.5(5.7)	**0.007**	23.2(4.9)	23.3(5.2)	0.973
Underweight	6.3	7.4	0.280	7.9	5.7	0.062	8.4	5.2	0.130	6.9	6.8	0.235	7.7	5.9	0.425
Normal weight	58.7	62.7		63.5	58		57.3	64		64	57.6		58.2	63.2	
Overweight	22.5	21.1		18.3	25.3		23.4	20.2		19.1	24.4		23.4	20.2	
Obese	12.6	8.8		10.3	11		10.8	10.5		10	11.2		10.6	10.7	
Physical activity															
Poor	17.6	17	0.980	19.6	15.8	0.270	15.7	18.6	**0.037**	16.3	17.9	0.570	13.3	19.8	0.276
Intermediate	74.7	75		75	74.8		80.1	70.6		77.8	73.2		78.3	72.7	
Ideal	7.7	8		5.4	9.5		4.2	10.8		5.9	8.9		8.4	7.5	
Menopause (% yes)	12.6	17.5	0.150	16.2	14.3	0.590	15.5	15.3	0.960	17.2	12.7	0.190	14.8	16.3	0.661

^a^ Results are reported as median (interquartile range) or percentage. Statistical analysis was performed using Chi-square test for bivariate or categorical variables and Mann–Whitney test for continuous variables. ^b^ Significant results are indicated in bold.

**Table 2:** Characteristics of women according to consumption of meat, dairy products, alcohol, and lipids ^a^.

Characteristics	Meat			Dairy Products			Alcohol			Unsaturated/Saturated Ratio		
	Poor	Ideal	*p*-Value ^b^	Poor	Ideal	*p*-Value ^b^	Poor	Ideal	*p*-Value ^b^	Poor	Ideal	*p*-Value ^b^
Age. years	43(13)	44(14)	**<0.001**	45(12)	42(15)	**0.012**	43(13)	45(15)	0.481	44(14)	44(13)	0.056
1st tertile (25–33 years)	39.0%	26.7%	**<0.001**	29.5%	36.2%	**0.029**	31.9%	36.5%	0.524	34.3%	31.4%	0.343
2nd tertile (34–46 years)	34.4%	34.3%		33.7%	35.0%		34.9%	32.4%		35.2%	33.5%	
3rd tertile (47–65 years)	26.6%	39.0%		36.8%	28.8%		33.2%	31.2%		30.5%	35.2%	
Educational level												
Low	38.2	33.1	0.054	34.2	37.1	0.582	37.1	30	0.174	31.7	39.7	**0.021**
Medium	45.6	44.5		46.8	43.3		44.6	47.1		46.2	43.9	
High	16.2	22.4		19	19.5		18.3	22.9		22.1	16.4	
Employment status (% unemployed)	57.2	53.3	0.253	53.9	56.7	0.424	56.8	49.4	0.084	52.4	58.2	0.093
Marital status (% living in couple)	49.5	51.8	0.621	52.4	49	0.426	51.8	45.2	0.223	48.5	52.5	0.352
Smoking status												
Never smokers	53.6	57.9	0.050	61	50.5	**0.006**	55.1	30.2	0.407	59.2	52.3	0.113
Former smokers	8.6	11.7		9.8	10.5		9.7	58		8.8	11.4	
Current smokers	37.9	30.5		29.3	39		35.2	11.8		32	36.3	
Use of supplements (% users)	13.2	17.9	0.134	16.9	14.1	0.372	14.7	18.3	0.371	15.8	15.1	0.836
Having children (% yes)	66.2	72	0.155	67.3	70.3	0.453	69.6	66.3	0.523	63.8	73.7	**0.013**
Number of children	2(2)	2(1)	0.991	2(1)	2(2)	0.122	2(1)	2(2)	0.954	2(2)	2(1)	0.814
Body Mass Index. kg/m^2^	23.4(5.7)	22.9(4.6)	0.774	23.3(4.9)	23.2(5.1)	0.331	23.14(4.7)	23.8(6)	0.211	23.05(5.1)	23.43(5)	**0.037**
Underweight	7.7	6	0.612	5.5	8.1	0.465	5.4	12.4	**0.006**	8.6	5	0.147
Normal weight	58.9	62.6		60.7	60.8		62.3	54.4		59.7	61.7	
Overweight	22	21.6		22.5	21.1		21.2	24.3		22.3	21.3	
Obese	11.5	9.8		11.3	10		11.1	8.9		9.4	12	
Physical activity												
Poor	19.8	15.2	0.501	16.9	17.8	0.866	17.5	16.7	0.986	16.1	18.3	0.741
Intermediate	72.7	76.8		75.8	73.6		74.7	75.6		76.8	73.3	
Ideal	7.6	8.1		7.2	8.6		7.9	7.7		7.1	8.4	
Menopause (% yes)	13	18	0.143	16.1	14.8	0.715	17.3	7.9	**0.030**	12.6	18	0.112

^a^ Results are reported as median (Interquartile range) or percentage. Statistical analysis was performed using Chi-square test for bivariate or categorical variables and Mann–Whitney test for continuous variables. ^b^ Significant results are indicated in bold.

**Table 3:** Characteristics of women according to adherence to Mediterranean diet ^a^.

Characteristics	Mediterranean Diet			
	Low	Medium	High	*p*-Value ^b^
Age, years	36.0 (17.0)	41.0 (20.0)	50.0 (25.0)	**<0.001**
1st tertile (25–33 years)	41.2%	29.5%	22.8%	**<0.001**
2nd tertile (34–46 years)	37.3%	34.5%	22.8%	
3rd tertile (47–65 years)	21.5%	36.0%	54.4%	
Educational level				
Low	44.6%	37.0%	25.4%	**<0.001**
Medium	40.7%	41.6%	52.9%	
High	14.6%	21.4%	21.8%	
Employment status (% unemployed)	57.9%	54.8%	53.2%	**0.017**
Marital status (% living in couple)	44.5%	56.6%	58.1%	**0.017**
Smoking status				
Never smokers	46.4%	59.4%	61.3%	**<0.001**
Former smokers	7.5%	7.1%	15.8%	
Current smokers	46.1%	33.5%	22.9%	
Use of supplements (% users)	14.7%	14.5%	29.0%	0.097
Having children (% yes)	65.1%	72.8%	74.2%	0.142
Number of children	2.0 (1.0)	2.0 (1.0)	2.0 (1.0)	0.275
Body Mass Index, kg/m^2^	26.30 (9.10)	23.34 (4.30)	23.05 (5.10)	**<0.001**
Underweight	2.5%	8.2%	9.7%	**<0.001**
Normal weight	54.3%	66.3%	61.5%	
Overweight	28.1%	18.3%	19.1%	
Obese	15.1%	7.2%	9.7%	
Physical activity				
Poor	13.1%	17.0%	19.2%	0.344
Intermediate	82.1%	71.6%	73.2%	
Ideal	4.8%	11.4%	7.6%	
Menopause (% yes)	11.6%	19.3%	20.8%	0.072

^a^ Results are reported as median (Interquartile range) or percentage. Statistical analysis was performed using Chi-square test for bivariate or categorical variables and Mann–Whitney test for continuous variables. ^b^ Significant results are indicated in bold.
